# Heattech clothing technology and potential for thermal injury in MRI

**DOI:** 10.1259/bjrcr.20220012

**Published:** 2022-10-05

**Authors:** Darren Hudson, Luis Abrantes

**Affiliations:** 1 MRI Clinical Lead, InHealth Group, High Wycombe, United Kingdom; 2 Head of Operations, South, InHealth Group, High Wycombe, United Kingdom

## Abstract

A patient attended for an MRI scan wearing Heattech thermal clothing. Following the scan, the patient experienced a heating and sunburn sensation over their back. Further investigation has highlighted one similar event to this internationally due to the clothing technology used. The intention of this report is to raise awareness of the potential for this clothing technology to cause thermal injury when worn within MRI, as well as to further emphasise the importance of assessing patient clothing prior to scanning.

## Clinical presentation

A male patient within their early 50s, with weight 65 kg and height 170 cm, presented for an MRI scan of their lumbar spine to assess for lower back and right leg pain with gluteal muscle wasting. A routine scan protocol, comprising of Sagittal T2, T1, T2 Fat sat and axial *T_2_* weighted sequences, was performed on a 1.5 T Siemens Aera Tim [204 × 24] with XJ gradients in normal SAR mode, totalling 10 min. Sequence parameters as detailed in Table 1. The scanner is housed within a relocatable unit containing scan room, control room, one patient changing room and one cannulation bay. The patient was screened for any contraindications prior to entering the scan room. Due to the weather on the day, he was noted to be wearing multiple layers of clothing; a thermal top, t-shirt, button-up shirt, jumper and coat. He was asked to remove the coat and jumper and there were no metallic fasteners on the other layers to cause concern. The patient was positioned feet first within the scanner using a 24-element Spine Direct Connect Matrix Coil integrated into the patient table, with his legs supported by a knee pad, ear protection fitted, and a call bell provided. The scan proceeded with no immediate cause for concern.

**Table 1.** Scanning parameters used

Plane/Sequence	Sag T2	Sag T1	Sag T2 FS	Ax T2
FOV	300	300	300	200
TR	3820	546	3660	3550
TE	107	11	93	112
Averages	2	2	3	2
Oversampling (%)	60	60	80	20
Phase matrix	269	269	230	230
Frequency matrix	384	384	384	384
Slice thickness (mm)	4	4	4	4
Slice gap (mm)	1	1	1	1
Flip angle	140	120	143	140
Turbo factor	17	4	15	15
BW	150	159	161	161
Gradient mode	Normal mode	Normal mode	Normal mode	Normal mode

FOV, field of view; TE, echo time; TR, repetition time.

## Investigations

**Figure 1.**
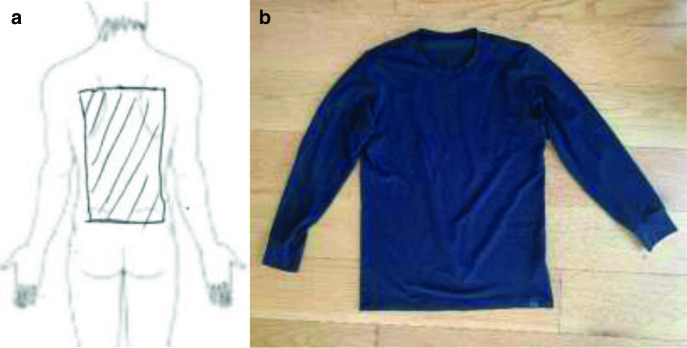
(a) Location of sensations and heating experienced by patient, and (b) photo of clothing item.

Two weeks following the appointment, a complaint was received from the patient. He described an experience of a heating sensation during the scan which was presumed to be normal and part of the image process. However, on returning to his car, he felt his back was still hot as if badly sunburned. This worsened over the following hours and medical attention was sought the next day from a minor injuries unit. Review confirmed no evidence of visible burns but that the back felt very hot to the touch (Figure 1a). The advice was to remain hydrated and take ibuprofen for the pain and that it should settle in a few days.

The patient continued to experience pain and an internal sensation of burning which was making it difficult to sit or lie, describing it like sunburn tearing the tissue inside their back when they moved or twisted. This time the patient saw his general practitioner (GP) who was unfamiliar with such an injury and prescribed Naproxen as an anti-inflammatory to help settle the discomfort whilst it healed.

Once made aware of the patient’s reported experience, an investigation was conducted. Only one report of a similar injury could be found in the existing literature relating to the same clothing technology,^1^ although others relating to metallic fibres within clothing have been recorded.^2,3^ The former report also makes reference to similar reports being made within Japan and to the manufacturer, suggesting a possible relationship between this clothing material and MRI (Figure 1b).

Internal assessment of patient set up confirmed positioning and scan mode had been adhered to correctly. The event was reported to Siemens who conducted an investigation on the system to ensure that there were no technical issues. The incident was then raised with the Medicines and Healthcare Products Regulatory Agency (MHRA) within the UK which provides oversight and guidance on management of MRI safety. There were no errors found with the scanner itself, the scans had been performed within the required restrictions, and the use of pads and a fan within the bore had been used to help mitigate any other heating risks. The length of time within the scanner was also short, at around 10 min, which would have further limited the amount of heating energy deposited within the patient.

5 months after the incident the patient reports still having a burning sensation, soreness, lack of elasticity and a feeling of tearing and tightness in his back. The patient was seen by a Consultant Neurologist due to these ongoing symptoms and reassurance was given to the patient that there was no indication of any neurological damage. An independent clinician of the patients has suggested that he may have suffered burning and damage to their fascia, which could potentially explain the ongoing pain, although this remains unproven and the cause for the patient symptoms cannot be 100% confirmed.

### Outcome, follow-up and discussion

Due to the nature of MRI, there is a heating effect associated with the technique as radiofrequency energy is applied to the body for the purposes of producing an image.^4^ Thermal-related injuries continue to be the most often reported incidents,^5^ perhaps because there is evident patient harm associated with these. They are mainly attributable to creation of conduction loops, near field effects, or conductive items being present within the bore.^5^ None of these appear to have been the cause in this case.

The risk of thermal injuries happening is primarily controlled through the design of the scanning equipment and whilst performing the examination.^6^ Use of normal mode specific absorption rate (SAR) level during scanning is advocated for routine use which limits the whole body SAR to 2 Watts/kg, which in turn limits the rise in whole body temperature to no more than 0.5°C.^7^ The indirect effects of heating are effectively managed through safe working practices,^6^ such as safe positioning (pads), preparation (clothing) and other adjuncts to encourage natural cooling of the body (bore fan, controlled ambient temperature).

The ongoing nature of the patient’s injury is extremely unusual. Symptoms could be linked to a secondary degree burn within the underlying dermal layers of the skin, which, whilst painful, should usually resolve after around 2 weeks.^8^ The exact nature or mechanism which could have caused such an injury is unclear. MRI-related radiofrequency burns are such that when non-contact related, they can be delayed in appearance and develop subcutaneously outwards. In this case, no visible delayed blistering to the epidermis was seen, nor prominent erythemal reddening.

An important component around preparation for MRI is how patients are dressed for the procedure. The intention is primarily around management of metallic components which could become a projectile or cause image artefacts. However, there is an important consideration too around the type of clothing worn, and number of layers, so as not to inhibit heat loss and cooling from the areas subjected to radiofrequency exposure.

This is where awareness of new clothing technologies is important as well as effective dialogue with patients around their clothing attire. It is very much a discussion between the staff and patient to ascertain what steps are needed to maintain safety and comfort, as some people feel the cold more than others when the room is kept to a controlled temperature.

In this case, whilst a coat and jumper were removed, three other layers remained (one of which was the thermal Heattech top). In the other reported case,^1^ the patient was scanned with four layers of clothing on which included the Heattech product. Each season brings with it its own challenges around managing patient clothing and requires careful consideration; summer shorts and t-shirts present issues around increased bare skin and the risk of conduction loops, whilst winter comes with the consideration over multiple layers that can inhibit natural body cooling processes.

As described in this case report, it is important to be aware of the potential effects of different clothing materials within MRI. As well as the aforementioned case with a patient wearing similar clothing materials, there have been cases reported around the use of specialist sportswear containing conductive fibres, such as silver thread, which have been known to cause an enhanced heating effect and cutaneous burning.^2,3^ Preference should perhaps be for more natural fibre clothing (cotton) which is often more breathable compared with more manmade materials, such as polyester or acrylic, which can inhibit heat loss and retain moisture around skin.

The manufacturers of Heattech use 38% Rayon, 31% Acrylic, 24% Polyester, 7% Spandex in their thin, lightweight clothing which is available in a range of types, not just undergarments. There are varying degrees by which they will retain heat (Table 2). The material uses synthetic man-made fibres which have been designed to retain heat and enhance insulation by using the moisture released by the body, thereby inhibiting natural cooling.

**Table 2.** Summary of Heattech clothing features

Features of Heattech		Heattech	Heattech extra warm	Heattech ultra warm
Heat generation	Water vapour from the body is absorbed by highly absorbent rayon. The energy from this moisture is converted to heat, giving the fabric itself a unique warming effect.			
Heat retention	Ultra-fine microfibres create a highly insulating layer of air around the body		1.5x thicker and warmer	2.25x thicker and warmer

https://www.uniqlo.com/uk/en/content/heattech-landing-men.html

Heating adjacent to any receive coil can be noted as part of the normal imaging process. The staff provided a call bell, in accordance with standard procedure, and explained that it should be used to call the attention of staff if necessary. It is also standard practice for staff to check in on patients in between sequences to ensure they are okay and to support the management of any issues raised by the patients during the MRI scan, such as feeling hot. On this occasion, staff were not alerted to any discomfort during the scan, with the warmth experienced not considered greater than expected or uncomfortable.

To conclude, this case report presents an atypical thermal-related injury subsequent to an MRI scan. It highlights a relatively new clothing material that the authors and employing organisation were not aware of but appears to have the potential to further inhibit natural cooling and lead to possible thermal-related side-effects. It serves to raise awareness of this product and of the need to carefully consider preparation for MRI examinations in order to minimise the risk for harm.

## Learning points

- Raising awareness of new clothing technologies which can inhibit heat loss and may lead to atypical thermal injuries to patients undergoing MRI
- Reiterates the importance of considering patient clothing, and the number of layers, when preparing for MRI scans
